# Work hours and the risk of hypertension: the case of Indonesia

**DOI:** 10.1186/s12889-024-20003-z

**Published:** 2024-09-12

**Authors:** Friska Aulia Dewi Andini, Adiatma Y. M. Siregar

**Affiliations:** 1 Center for Economics and Development Studies, Department of Economics, Faculty of Economics and Business, Universitas Padjadjaran, Jl. Cimandiri no. 6-8, Bandung, West Java 40115 Indonesia; 2 Department of Economics, Faculty of Economics and Business, Universitas Padjadjaran, Jl. Raya Bandung Sumedang KM.21, Sumedang, West Java 45363 Indonesia

**Keywords:** Work hours, Hypertension, Indonesia, IFLS

## Abstract

**Background:**

Individuals working excessive hours is a worldwide phenomenon. In Indonesia, over 32 million people work more than 40 h per week, contributing to around 26% of the workforce. Excessive working may affect health, increasing the risk of cardiovascular diseases such as hypertension. Hypertension affected around 34% of Indonesian adults, approximately 63.3 million people and led to about 427,000 deaths in 2018, and the prevalence remains high at 29.2% in 2023. This study aims to analyze the relationship between work hours and the risk of hypertension among working individuals in Indonesia.

**Methods:**

This study used a pooled cross-sectional data from the Indonesian Family Life Survey (IFLS) wave 4 (2007) and wave 5 (2014) and performed a logit regression analysis to examine the likelihood of a working individual having hypertension based on the individual’s work hours. A dummy variable of hypertension is created based on the result of blood pressure measurement. The sample consists of 22,500 working individuals in Indonesia. This study controlled for job characteristics, sociodemographic status and health-behavioral risk factors such as BMI and smoking behavior, and performed additional regression analyses for alternative models to check for robustness.

**Results:**

Our findings showed that there is a higher probability of having hypertension for workers who work longer hours by 0.06% points for each additional hour of work (*p* < 0.01). Other factors such as physical activity and smoking behavior have also been demonstrated to be significantly correlated to the risk of hypertension.

**Conclusions:**

This study revealed a positive relationship between work hours and hypertension. Although this study cannot suggest causality, the strongly significant correlation may provide an idea and an overview regarding the risk of hypertension among working individuals in Indonesia. The Indonesian government could consider conducting further studies to implement and promote flexible working arrangements initiatives and incentive programs to improve workers’ health outcomes.

## Background

According to a 2023 report by International Labour Organization [1], around 35% of the global workforce work excessively in 2019. The share of those employed that work excessively (49 or more hours per week) in Mexico is around 27.63%, Vietnam 27.23%, Colombia 24.15%, Costa Rica 23.24%, and Indonesia 25.76%—which is equivalent to approximately 35 million people in 2022 [2]. As a middle-income country, Indonesia tends to have people working longer hours compared to those in high-income countries [3]. Indonesian Bureau of Statistics (BPS) noted that for the past few years, even during the pandemic of Covid-19, the number of people working excessive hours (more than 40 h per week) has always been over 32 million people or around 26% [4, 5]. This definition of excessive work hours may differ across countries as it also differs from the ILO’s. However, the United States, Japan, and South Korea have the same standard for maximum normal working hours per week in general [6–8].

Although it is common for workers to work overtime, working long hours may adversely affect health. The health implications may vary due to several factors such as job characteristics, socioeconomic status, and health condition. Besides affecting mental health, working excessive hours can increase the risk of cardiovascular diseases, including hypertension [3].

Hypertension is a major cause of premature death, and one of the global targets for noncommunicable diseases is to reduce the prevalence of hypertension by 33% between 2010 and 2030 [9]. However, the global prevalence trend of hypertension has increased significantly over the past two decades, with a rate of 165.11 prevalent cases per 100.000 in 1999 and continued increasing until 240.36 prevalent cases per 100.000 in 2019 [10]. The prevalence of hypertension among Indonesian adults has increased from 25.8% in 2013 to 34.1% in 2018 [11] amounting to approximately 63.3 million people, with around 427,000 deaths [12]. However, the latest survey conducted by the Indonesian Ministry of Health revealed that the prevalence of hypertension has then decreased to 29.2% in 2023. Although the country observed a decrease, the prevalence remains high [13].

Studies have been conducted to analyze the relationship between working hours and blood pressure. However, findings on association between working hours and hypertension have been varied. Some suggest that excessive working or long working hours could potentially serve as a work-related risk factor for high blood pressure or hypertension [14–16]. A study by Guner [17] found that working more hours reduces the probability of hypertension for both men and women. The lack of consistency on research findings could be attributable to certain constraints regarding someone’s socioeconomic, demographic, and health status.

Excessive work hours may lead to exhaustion, notably due to sleep deprivation [18], which has been associated to the incidence of hypertension. Meanwhile, the recommended duration of sleep for adults are 7–9 h per day [19]. Some studies have found an increased risk of hypertension for individuals with short sleep duration [20–22]. Conversely, a study by Zhang et al. [23] found higher odds of hypertension associated with long sleep duration among Chinese rural population. However, a meta-regression analysis of around 5 million participants found no significant relationship between long sleep duration and the incidence of hypertension [24].

To see the relationship between working hours and the risk of hypertension from one perspective, that is increasing physical activity—long working hours might actually reduce the probability of someone having hypertension [25, 26]. However, stressful working conditions may also contribute to a higher risk of hypertension. In a scoping review by Kumbu et al. [27] covering workers in Sub-Saharan Africa, it has been found that job stress is positively and significantly associated with hypertension. Hence, factors relating to job characteristics, such as physical activity and job stress, might also influence the risk of hypertension.

Some countries limit overtime hours, that is additional hours worked in excess of the normal work hours. Japan and South Korea limit their daily overtime hours to 4 (four) hours a day, and their weekly overtime hours to 15 (fifteen) hours and 12 (twelve) hours, respectively, with normal working hours of 8 (eight) hours a day and 40 (forty) hours a week [7, 8]. In another case, the United States does not impose a limit on overtime hours for employees aged 16 or above, although certain states such as California and Maine have different regulations [28–30]. In Indonesia, based on the Government Regulation No. 35 of 2021 Article 26 [31], workers’ overtime work can only be done for a maximum of 4 (four) hours in a day and 18 (eighteen) hours in a week, with normal working time provision of 40 h a week. There is an increase of one hour per day and four hours per week in workers’ overtime allowed compared to the old regulations of Manpower Act No. 13 of 2003 [32]. Notably, this might lead to an increase in overtime hours worked by workers. Given the potential impact of increasing work hours on workers’ health, this study aims to analyze the effect of work hours on the risk of hypertension among working individuals in Indonesia.

## Methods

### Data

The data used in this study originate from the Indonesian Family Life Survey (IFLS) wave 4 (2007) and wave 5 (2014) [33, 34]. The IFLS is a longitudinal survey, first conducted in 1993, with sample representing about 83% of Indonesian population. The purpose of this survey is to provide data for studying behaviors and outcomes. It encompasses a substantial amount of information collected at the individual and household levels, containing social, demographic, economic and non-economic characteristics, including health status [35]. The analysis of this study used a pooled cross-sectional data consisting a sample size of 22,500 working individuals aged 15 or older who accommodate measurement of blood pressure when the surveys were conducted.

### Variables

This study used measured hypertension as the dependent variable. The main independent variable in this study is work hours per week and the rest of the variables presented in the model are control variables. The data set included sociodemographic characteristics and health behavioral-risk factors: body mass index (BMI) and smoking behavior. These variables were used in accordance to previous studies [14, 16]. This study also included work-related variables, particularly type of employment and job characteristics that weren’t present in the previous studies. In addition, this study also controlled for survey wave, sociodemographic variables including education, per capita expenditure, residence, gender, marital status and age. Education level was the highest education level the individual attended and was classified into 5 categories: (1) no schooling, (2) elementary school, (3) lower secondary school, (4) upper secondary school, (5) higher education; with the first group, no schooling, as the reference group. Table 1 provides a further description of some variables in the model.

**Table 1:** Variable description

Variables	Description
Measured hypertension	A dummy variable with 1 indicating hypertension and 0 indicating no hypertension. Hypertensive if blood pressure measurement which is conducted twice results: systolic = 130 or higher; and/or diastolic = 80 or higher
Work hours per week	“Normally, what is the approximate total number of hours you work per week?”
Primary job characteristics: Physical activity Lifting heavy loads Stooping, kneeling, crouching Using computers Stress	“My job requires/involves (primary job characteristic)” 0 if the job requires little to no activity of the primary job characteristic 1 if the job requires moderate to high activity of the primary job characteristic
Body mass index (BMI)	Individual’s body mass index
Smoking behavior	Never smoker if never smoke at all Former smoker if ever smoke and has totally quit Current light or moderate smoker if reports consuming < 20 cigarettes per day Current heavy smoker if reports consuming 20 or more cigarettes per day
Type of employment	Determined based on the definition by BPS: formal if the individual has a status of laborer/worker/employee and those who do business with permanent laborers; or informal if otherwise
Per capita expenditure	Individual’s per capita expenditure in natural logarithmic form

### Analysis

In analyzing the relationship between hypertension and work hours, this study employed a logit regression model with standard deviation clustered at individual level to measure the contribution of work hours to the probability of a working individual having hypertension. To check for robustness, this study also employed an ordinary least squares (OLS) regression and another logit regression analysis.

Logit regression models the probability of a binary outcome (i.e., risk of hypertension: hypertensive or not) based on one or more predictor variables. The primary focus is on understanding how changes in the predictors affect the likelihood of the outcome occurring. To interpret the effects of predictors, average marginal effects are used to describe the average change in the predicted probability of the outcome for a one-unit change in the predictor [36]. The logit regression equation for the main model in this study is as follows:

$$\:P(hypertension=1)=\:\alpha\:+{\beta\:}_{1}workhours+{\beta\:}_{i}{X}_{i}+\epsilon\:$$

As the first alternative to check for robustness, an OLS regression with robust standard errors was performed to find the hyperplane that best fits the data by minimizing the sum of the squared differences between the observed values and the values predicted by the linear model. This minimization leads to the OLS estimates for the coefficients, which represent the best linear unbiased estimators (BLUE) under the Gauss-Markov assumptions [36]. This model assumed linearity and used mean arterial pressure (MAP) as the continuous form of blood pressure instead of a dummy hypertension for the dependent variable. MAP is calculated as: MAP = Diastolic Pressure + 1/3(Systolic Pressure – Diastolic Pressure) [37]. Since the IFLS provides two blood pressure measurements for each individual, this study used the average of the MAP values, labeled as average MAP. The OLS regression equation is as follows:

$$\:{Average\:MAP}_{i}=\alpha\:+{\beta\:}_{1}workhours+{\beta\:}_{i}{X}_{i}+\epsilon\:$$

In the second alternative model, this study used a dummy variable of excessive work hours instead of a continuous form for the main independent variable. Based on the regulation of normal work hours in Indonesia, those who work more than 40 h per week are considered as individuals with excessive work hours. The logit regression equation is as follows:

$$\:P\left(hypertension=1\right)=\alpha\:+{\delta\:}_{0}excessivework+{\beta\:}_{i}{X}_{i}+\epsilon\:$$

## Results

### Descriptive statistics

To provide an overview of the data used in this study, Table 2 shows the descriptive statistics of the variables for the entire sample of working people in Indonesia. The upper part of the table shows the mean and standard deviation of the continuous variables and the lower part shows the percentage of the indicator variables.

**Table 2:** Descriptive statistics

Variable	Mean	Std. Dev
Work hours per week	41.69	21.76
Average MAP	95.15	12.99
BMI	25.06	67.70
Per capita expenditure	738,453	759,981
Age	36.30	13.72
Observations	22,500	
**Percentage (%)**		
Hypertension	29.20	
Physical activity	55.24	
Lifting heavy loads	31.54	
Stooping, kneeling, crouching	50.37	
Using computers	8.28	
Formal	41.05	
Stress	7.42	
Smoking behavior		
Never smoker	58.97	
Former smoker	2.88	
Current light to moderate smoker	32.86	
Current heavy smoker	5.28	
Male	53.35	
Married	71.77	
Rural	47.52	
Education level		
No schooling	5.87	
Elementary school	33.75	
Lower secondary school	18.40	
Upper secondary school	29.31	
Higher education	12.68	
Observations	22,500	

Source: IFLS, Author’s calculation

Most Indonesians work around 42 h per week (s.d. 22 h). There are 6,570 people who are objectively measured as having hypertension, amounting to about 29%. Regarding job characteristics, the percentage of people with jobs requiring moderate to high physical activity is approximately 55%; meanwhile, the percentage of those with jobs involving moderate to high level of stress is approximately 7%. Our sample has to some extent reflect the labor force participation rate in Indonesia as compared to Indonesian National Labor Force Survey 2021 [38] as the result shows that the workforce is dominated by male workers at approximately 53%.

Body mass index variable shows that average working individuals in Indonesia have a BMI of 25, which is categorized as overweight (s.d. 68). Smoking behavior among working people shows that the largest portion, almost 59%, comprises those who never smoke with current light to moderate smokers coming second at nearly 33%. Among all, most have an education level of elementary school and upper secondary school, that is 34% and 29%.

**Table 3:** Prevalence of hypertension across age groups and genders

	Num. with hypertension	Total	Prevalence (%)
Age group			
15–24	920	4,926	18.68
25–54	4,413	15,076	29.27
55–64	750	1,588	47.23
> 64	487	910	53.52
Gender			
Male	3,314	12,003	27.61
Female	3,256	10,497	31.02

Source: IFLS, Author’s calculation

The prevalence of hypertension is calculated as the proportion of individuals with hypertension within age group and based on gender. Table 3 shows that the prevalence of hypertension increases with advancing age. The highest prevalence is observed in the age group > 64 at around 54%. It also shows that the prevalence of hypertension is found higher among female compared to male workers.

### Regression analysis

Table 4 shows the estimation result of the logit model. The findings indicate that at the mean of 42 h per week, controlling for sociodemographic and health behavioral-risk factors of hypertension, an hour increase in work hours per week of an individual will increase the probability of that individual having hypertension by 0.06% points (*p* < 0.01). In addition, several control variables showed significant associations with the risk of hypertension as presented in the table at varying significance levels.

**Table 4:** Logit regression result (dependent variable: measured hypertension)

Variable	Coefficient	Marginal effect
Work hours per week	0.0031*** (0.0007)	0.0006*** (0.0001)
Physical activity	-0.0931*** (0.0365)	-0.0168*** (0.0065)
Lifting heavy loads	-0.0931** (0.0409)	-0.0165** (0.0073)
Stooping, kneeling, crouching	0.0428 (0.0359)	0.0076 (0.0064)
Using computers	-0.1088** (0.0652)	-0.0230** (0.0116)
Stress	-0.1088* (0.0610)	-0.0193* (0.0108)
Formal	0.0924** (0.0364)	0.0164** (0.0001)
BMI	0.0005** (0.0002)	0.0001** (0.0000)
Smoking behavior (ref: never smoker)		
Former smoker	-0.1532 (0.1036)	-0.0277 (0.0183)
Current light or moderate smoker	-0.3588*** (0.0507)	-0.0626*** (0.0087)
Current heavy smoker	-0.1443* (0.0825)	-0.0262* (0.0147)
Male	0.2416*** (0.0469)	0.0429*** (0.0083)
Married	0.0175 (0.0410)	0.0429 (0.0073)
Rural	-0.0889*** (0.0347)	-0.0158*** (0.0062)
lnPCE	-0.1142*** (0.2622)	-0.0212*** (0.0047)
Age	-0.0599*** (0.0072)	-0.0106*** (0.0013)
Agesq	0.0013*** (0.0001)	0.0002*** (0.0000)
Education level (ref: no schooling)		
Elementary school	0.1614** (0.0789)	0.0293** (0.0141)
Lower secondary school	-0.0836 (0.0871)	-0.0151 (0.0154)
Upper secondary school	-0.0677 (0.0871)	-0.0116 (0.0154)
Higher education	-0.0795 (0.0976)	-0.0124 (0.0172)
Survey wave 5 (2014)	1.3233*** (0.0398)	0.2349*** (0.0066)
_cons	0.0886 (0.3468)	
Wald chi2(22)	2163.32	
Prob > chi2	0.0000	
Observation	22,500	22,500

Note: *** *p* < 0.01, ** *p* < 0.05, * *p* < 0.1

Source: IFLS, Author’s calculation

An individual with a job involving moderate to high level of physical activity has a 1.68% points lower probability of having hypertension compared to those with little to no physical activity in their job (*p* < 0.01). Furthermore, lifting heavy loads and using computers are found to be negatively related with hypertension (*p* < 0.05). Stress level involved in individuals’ job is also found to be negatively related (*p* < 0.10). Being a current smoker is also found to be associated with lower risk of hypertension (*p* < 0.01; *p* < 0.10). Additionally, the result shows that a male worker relatively has a higher probability of having hypertension than a female worker by 4.29% points (*p* < 0.01).

**Table 5:** OLS regression result (dependent variable: average MAP)

Variable		Coefficient	95% Confidence Interval
Work hours		0.0072*	-0.0003–0.0147
Physical activity		-0.5937***	-0.9695 – (-0.2179)
Lifting heavy loads		-0.4699**	-0.8749 – (-0.0650)
Stooping, kneeling, crouching		0.0509	-0.3090–0.4108
Using computers		-0.5153*	-1.1108–0.0803
Stress		-0.2938	-0.8591–0.2714
Formal		0.4293**	0.0706–0.7880
BMI		0.0029**	0.0001–0.0058
Smoking behavior (ref: never smoker)			
Former smoker		-0.3400	-1.4326–0.7527
Current light or moderate smoker		-2.4885***	-2.9684 – (-2.0086)
Current heavy smoker		-1.3892***	-2.1893 – (-0.5890)
Male		3.5404***	3.0678–4.0129
Married		-1.4207***	-1.8201 – (-1.0214)
Rural		-0.0988	-0.4452–0.2475
lnPCE		0.4709***	0.2109–0.7309
Age		0.5409***	0.4757–0.6060
Agesq		-0.0025***	-0.0033–0.0017
Education level (ref: no schooling)			
Elementary school		0.0884	-0.7984–0.9752
Lower secondary school		-1.1548**	-2.0970 – (-0.2127)
Upper secondary school		-1.4848***	-2.4322 – (-0.5373)
Higher education		-2.0790***	-3.1193 – (-1.0387)
Survey wave 5 (2014)		-0.0778	-0.4614–0.3058
_cons		74.0914	70.6151–77.5677
Prob > F		0.0000	
R-squared		0.1417	
Observation		22,500	22,500

Note: *** *p* < 0.01, ** *p* < 0.05, * *p* < 0.1

Source: IFLS, Author’s calculation

Table 5 shows the logit regression result for the alternative model which used continuous blood pressure as the dependent variable. It further confirms the finding of the positive and significant relationship between work hours and higher blood pressure (*p* < 0.10).

Table 6 presents the logit regression result of the second alternative model, using dummy excessive work hours as the independent variable. The result shows that excessive work hours are positively and significantly related to higher risk of hypertension (*p* < 0.01), verifying the main finding of this study. It also serves as a robustness check, ensuring that the estimation of the main model used in this study is robust where the core variables are valid to interpret [39]. Furthermore, the odds ratios and confidence intervals are presented in Table 7.

**Table 6:** Logit regression result (dependent variable: measured hypertension)

Variable	Coefficient	Marginal effect
Excessive work hours	0.0845*** (0.0329)	0.0150*** (0.0058)
Physical activity	-0.0941*** (0.0365)	-0.0167*** (0.0065)
Lifting heavy loads	-0.0925** (0.0409)	-0.0164** (0.0073)
Stooping, kneeling, crouching	0.0398 (0.0359)	0.0071 (0.0064)
Using computers	-0.1273* (0.0652)	-0.02261* (0.0116)
Stress	-0.1023* (0.0610)	-0.0182* (0.0108)
Formal	0.0910** (0.0366)	0.0162** (0.0065)
BMI	0.0005** (0.0002)	0.0001** (0.0000)
Smoking behavior (ref: never smoker)		
Former smoker	-0.1532 (0.1036)	-0.0277 (0.0183)
Current light or moderate smoker	-0.3563*** (0.0506)	-0.0622*** (0.0087)
Current heavy smoker	-0.1402* (0.0826)	-0.0254* (0.0147)
Male	0.2412*** (0.0469)	0.0428*** (0.0083)
Married	0.0213 (0.0410)	0.0038 (0.0073)
Rural	-0.0947*** (0.0347)	-0.0168*** (0.0062)
lnPCE	-0.1142*** (0.2622)	-0.0203*** (0.0047)
Age	-0.0594*** (0.0072)	-0.0105*** (0.0013)
Agesq	0.0013*** (0.0001)	0.0002*** (0.0000)
Education level (ref: no schooling)		
Elementary school	0.1614** (0.0789)	0.0295** (0.0141)
Lower secondary school	-0.0836 (0.0871)	-0.0146 (0.0154)
Upper secondary school	-0.0677 (0.0871)	-0.0119 (0.0154)
Higher education	-0.0795 (0.0976)	-0.0139 (0.0172)
Survey wave 5 (2014)	1.3205*** (0.0398)	0.2345*** (0.0065)
_cons	0.0886 (0.3468)	
Wald chi2(22)	2154.45	
Prob > chi2	0.0000	
Observation	22,500	22,500

Note: *** *p* < 0.01, ** *p* < 0.05, * *p* < 0.1

Source: IFLS, Author’s calculation

**Table 7:** Work hours and hypertension among working individuals

	Odds Ratio (OR)	95% Confidence Interval	*p*-value
Work hours	1.0031	1.0017–1.0045	0.000
Excessive work hours	1.0881	1.0201–1.1607	0.010

Odds ratio (95% CI) for hypertension were estimated using logit regression, controlling for the variables as presented in Tables 4 and 6

## Discussion

On average, Indonesians work normal hours, with the majority required to do moderate to high physical activity in their workplace. Additionally, individuals with jobs requiring bad posture (stooping, kneeling, crouching) and lifting heavy loads contribute to a noticeably large portion. This aligns with the fact that the majority of Indonesian are informal workers [40], as also shown in Table 2. The average weekly work hours, as shown in Table 2, are approximately 42 h, which reflects the more recent data published by Indonesian Bureau of Statistics (BPS) that noted an average of 42 h a week in August 2023. There has been an increase of 2.44% compared to February—an average of 41 h a week, initially [5].

The result of this study, as shown in Table 4, indicates a significant and positive relationship between hypertension and work hours, meaning that the probability of having hypertension is higher for workers who work longer hours. This finding is in line with other studies that underscore that longer work hours are associated with a higher probability of having hypertension [14, 16, 41]. The alternative models as presented in Tables 5 and 6 further confirm this relationship, where longer work hours are associated with higher blood pressure which is also found in a recent study [42]. Various hypotheses have been proposed to explain the relationship between long working hours and the risk of having cardiovascular diseases. Longer work hours could cause sleep deprivation, increased stress-level, and less time to recover, which has been shown to increase the risk of hypertension [20–22, 43, 44]. Although this study performed a robustness check and the variables of primary interest show a consistent and robust estimation of correlation, the analysis cannot suggest a causality effect [39].

According to the International Labour Organization, the maximum standard working time is eight hours per day and 48 h per week, as the ILO Hours of Work (Industry) Convention (No. 1) of 1919 has introduced it as an international norm [45]. In some exceptional cases, working overtime is allowed, as long as the total working time is no higher than 10 h per day and no higher than 56 h per week. In Indonesia, overtime work hours can be done for a maximum of four hours per day and 18 h per week with a normal working time provision of 40 h a week—totaling a maximum of 58 h per week. This number is two hours higher than the international standard.

It is not uncommon for countries to have maximum working hours shorter than the international standard. In the United Kingdom, an initiative of a shorter workweek—4 days amounting to 32 h per week, had most businesses report a reduction in employees’ stress and an increase in their well-being [46]. However, a lot of Indonesian workers, especially in the manufacturing industry, are low-skilled labors and reducing work hours may impose challenges such as lower productivity and underutilization of labor [47]. Having similar characteristics, Indonesia may adopt the initiative of the flexible work arrangement of Philippines which allows employers and employees to make an agreement regarding work hours, work days, and work week [48]. This is meant to encourage employees’ work-life balance and also benefit the employers from increased productivity.

Some domestic employers utilize flexible working arrangements, which has been proven to be successful in the case of L’Oréal Indonesia [49]. Therefore, the government may facilitate and promote the initiative through regulations. Types of flexible work arrangements that are suitable for the family-oriented Indonesian culture would be telecommuting and flextime—where workers work normal hours at their workplace with preferred arrival and departure time. While telecommuting might improve family-time (e.g., work from home), it might lead to longer work hours if one could not set proper boundaries [50]. Further research is recommended as determining the effect of telecommuting among Indonesian workers is necessary to provide insights for the government’s decision-making. The Indonesian government would benefit from more research and data collection regarding working conditions and health outcomes which can inform evidence-based policy-making and initiatives. It may analyze the implications of its current standard of working hours and the best strategies to promote workers’ health. The government may also provide incentives to companies that implement measures to reduce overtime work and promote employee well-being. Additionally, knowing that the prevalence of hypertension is higher for older individuals as shown in Table 3, which is also found in a previous study [51], the government might benefit from further studies focusing on the risk of hypertension for middle-aged and older working individuals and whether they need any specialized approach of risk mitigation.

In addition, as the results suggest, several control variables have significant associations with the risk of hypertension. This study observed some contrary findings to some previous studies. While most evidence suggests a positive relationship between occupational stress and hypertension, this study found a negative association (*p* < 0.10). This can be explained by considering several personal variables that might mediate this relationship. Individuals with higher self-perceived capabilities, self-confidence, and effective coping mechanism are often better equipped to handle stress. These personal attributes can buffer the negative effects of stress, allowing individuals to manage their stress and prevent it from translating into physiological consequences such as hypertension [52]. The results also showed a lower probability for current smokers compared with individuals who never smoke, contrary to some previous studies [53, 54]. However, this result aligns with a finding by Li et al. [55], with the attributable cause being the long-term effects of smoking which often do not show up until later in life [56]. Furthermore, although this study found a higher probability of having hypertension for male workers and the disease is also known to be more likely in males [57], this study also observed the contrary as presented in Table 3 where there is a higher prevalence of hypertension among females relative to males. The possible cause is that females experience a much rapid rise in blood pressure beginning in their third decade of life and the prevalence of hypertension increases with age [58]. The study also mentioned that sustained vascular influence of hypertensive conditions of pregnancy, interactions between the renin-angiotensin-aldosterone system and sex hormones, or even psychosocial gendered issues such as socioeconomic deprivation may be responsible for these blood pressure trajectories.

While this study provides valuable insights regarding the relationship between working hours and the risk of hypertension among working individuals in Indonesia, it is important to acknowledge its limitations. The sample used in this analysis may not fully capture the true prevalence of hypertension among the Indonesian workforce, potentially leading to an overestimation which might result from the measurement of blood pressure that was conducted only in one same day. Furthermore, the analysis in this study might not estimate the causal effect of work hours on hypertension, taking into account the possibility of reverse causality where individuals’ decision regarding their work hours might be attributed to their health status. Further studies are needed to establish causality. Moreover, this study did not include other source of stressors, such as commuting, due to the information being unavailable. Despite these limitations, this study offers a comprehensive examination regarding the covered topic by utilizing the available data and alternative models to attempt for more robust results.

## Conclusions

This study found a significant and positive relationship between work hours and hypertension, meaning that longer work hours are associated with a higher risk of hypertension. Possible mechanisms are incidence of sleep deprivation, increased stress-level and less time to recover. Although this study cannot suggest causality, the strongly significant correlation may provide an idea and an overview regarding the risk of hypertension among working individuals in Indonesia. The Indonesian government, after conducting further studies, may adopt initiatives such as flexible working arrangements into its regulation and incentive provisions to promote workers’ health outcomes in the presence of the challenges the country may face in regulating work hours.

## Data Availability

The data used in this study are freely available in the RAND repository, https://www.rand.org/well-being/social-and-behavioral-policy/data/FLS/IFLS.html.
